# Three-Dimensional Copper Foil-Powder Sintering Current Collector for a Silicon-Based Anode Lithium-Ion Battery

**DOI:** 10.3390/ma11081338

**Published:** 2018-08-02

**Authors:** Jin Long, Huilong Liu, Yingxi Xie, Weijin Tang, Ting Fu, Yong Tang, Longsheng Lu, Xinrui Ding, Xingxian Tang

**Affiliations:** 1 State Key Laboratory of Pulp and Paper Engineering, South China University of Technology, Guangzhou 510640, China; longjin@scut.edu.cn; 2 Key Laboratory of Surface Functional Structure Manufacturing of Guangdong Higher Education Institutes, School of Mechanical & Automotive Engineering, South China University of Technology, Guangzhou 510640, China; huiloongliu@gmail.com (H.L.); tang0703@foxmail.com (W.T.); ytang@scut.edu.cn (Y.T.); meluls@scut.edu.cn (L.L.); dingxr@scut.edu.cn (X.D.); xingxian.tang@foxmail.com (X.T.); 3 Key Laboratory of Metallurgical Equipment and Control Technology, Wuhan University of Science and Technology, Wuhan 430081, China; futing1234gh@163.com

**Keywords:** lithium-ion battery, current collector, foil-powder sintering, silicon, cycle performance

## Abstract

In this work, we propose a facile method for manufacturing a three-dimensional copper foil-powder sintering current collector (CFSCC) for a silicon-based anode lithium-ion battery. We found that the CFSCC is suitable as a silicon-based paste electrode, and the paste-like electrodes are commonly used in industrial production. Compared with flat current collectors, the CFSCC better constrained the silicon volume change during the charging-discharging process. The capacitance of electrodes with CFSCC remained as high as 92.2% of its second cycle after 40 cycles, whereas that of electrodes with a flat current collector only remained at 50%.

## 1. Introduction

Silicon (Si) has attracted significant attention as a next-generation anode material for lithium-ion (Li-ion) batteries due to its theoretically superior specific capacity of 3578 mA∙h/g for the Li_15_Si_4_ phase at room temperature [1], which is about 10 times higher than that of carbon-based materials (~370 mA·h/g) [2]. However, conventional Si anodes are still limited in practical applications because Si exhibits a severe volume change (~300%) during lithiation and delithiation [3]. This effect can lead to the loss in electrical contact between active materials by mechanical fracture, and rapid capacity fading occurs during electrochemical cycling. To improve its performance, researchers have tried different silicon materials and structures, such as Si/carbon (C) hybrid nanostructures [4], silicon thin film [5], silicon nanowires [6], metallic coating [7], Si/TiSi_2_ heteronanostructures [8], and metallic foam [9]. These methods have been applied to Li-ion batteries to achieve better cycle performance. However, physical vapor deposition, multi-step electric deposition, electric beam etching, or multi-step chemical reaction have all been used, which have low productivity and are expensive in commercial applications. In this work, we propose a facile method for manufacturing a three-dimensional (3D) copper foil-powder sintering current collector (CFSCC) for Si-based anode Li-ion batteries. The CFSCC is suitable for Si-based paste electrodes, which are inexpensive, and the paste-like electrode is commonly used in industrial production. We found that the CFSCC significantly improved the cyclic performance of the Si-based electrode and reduced the fractures in the electrode.

## 2. Experimental

### 2.1. Materials and Methods

**Figure 1:**
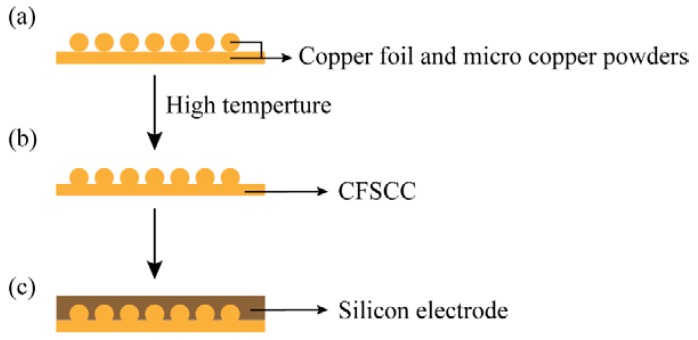
The battery fabrication process. (**a**) Copper foil and micro powder. (**b**) Copper foil-powder sintering current collector (CFSCC). (**c**) Silicon electrode pasted on CFSCC.

The fabricating process is schematically shown in Figure 1. A 30-μm thick copper foil and different sizes of micro copper powders (99.95% purity) were used to fabricate the copper current collector. First, one layer of micro copper powder was dispersed onto the copper foil surface with ultrasonic vibration. Then, the copper was heated to 950 °C and maintained at the same temperature for 3 h in a hydrogen atmosphere. After the copper cooled down, the micro copper powders were sintered into the copper foil, as shown in Figure 1b.

To assemble the half-cell battery, a mixture of silicon nanoparticle (300 nm, Shanghai ST-NANO Science & Technology Co., Ltd., Shanghai, China), acetylene black and polyvinylidene fluoride (Hefei Ke Jing Materials Technology Co., Ltd., Hefei, China) was used as the anode (weight ratio 7:2:1). The silicon electrode was 100 μm thick (Figure 1c). Lithium metal foil was used as the cathode. A polypropylene film (Celgard 2400, Celgard Inc., Charlotte, NC, USA) was used as the separator. Lithium hexafluorophosphate (1 M) was dissolved in ethylene carbonate and dimethyl carbonate (volume ratio 1:1) was used as the electrolyte (Samsung Corp of South Korea, Seoul, Korea). All chemicals and reagents were obtained commercially and used directly without further purification. The materials were assembled in a CR2025-type cell.

### 2.2. Characterization

The cyclic charge/discharge test (cyclic performance, coulomb efficiency and voltage-capacity profile) was conducted in the range of 0.02 and 1.5 V at the current density of 0.2 mA/cm^2^ on a commercial battery testing system (LAND CT2001A, Wuhan LAND electronics Co., Ltd., Wuhan, China). The cross-sections of sintered joints in CFSCC with different sizes of micro copper powders were prepared by wire electrical discharge machining (Wire EDM, Suzhou Baoma Corp., Guangzhou, China) and observed by a three-dimensional super depth optical microscope (VH-Z100R, Keyence Corp., Osaka, Japan). The surface morphology of the electrodes was characterized using a field emission scanning electron microscope (SEM, LEO 1530 VP, 5 kV, Germany).

## 3. Results and Discussion

### 3.1. Foil-Powder Sintering

**Figure 2:**
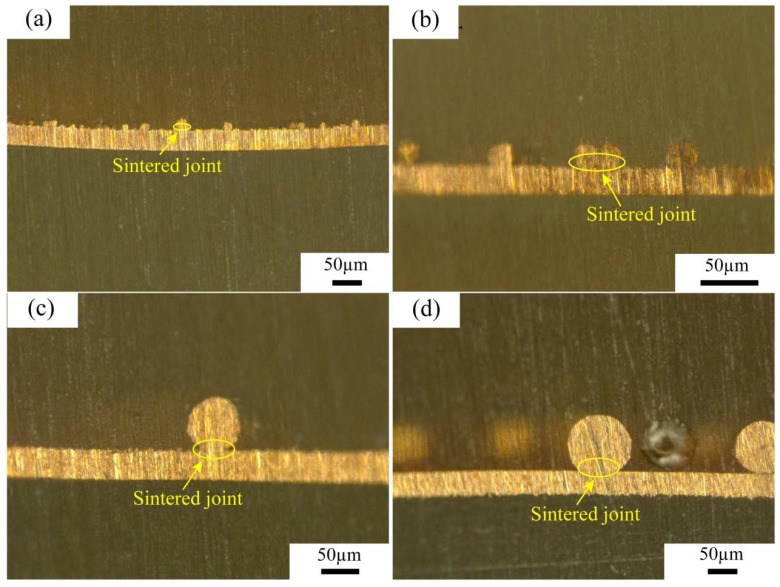
Cross-sectional optical images of sintered joints in CFSCC with different sizes of micro copper powders: (**a**) 0–25 μm, (**b**) 25–50 μm, (**c**) 50–75 μm, and (**d**) 75–100 μm.

Figure 2 shows the cross-sectional optical images of sintered joints in CFSCC with different sizes of micro copper powders. In this experiment, the sintering temperature was below the copper melting point (1085 °C). Some researchers studied the mechanics of the sintering process. Grupp et al. [10] reveals that particles in early stages of sintering not only roll with respect to their interparticle contacts, but also revolve at a greater angle around their own centers, even if they are firmly bonded to adjacent particles. Thus, during the sintering process, the kinetic energy of copper molecular is high, whereas the surface energy of the interface is low. Both the grain boundary diffusion and the surface diffusion occurred at a relatively high rate to form the joint neck. As the sintered joints enlarge, the surface energy of interface increased with decreasing sintering force. The surface energy attained balance with a stable joint neck curvature. After the copper cooled to room temperature, the sintered joints were formed.

### 3.2. Electrochemical Performance

**Figure 3:**
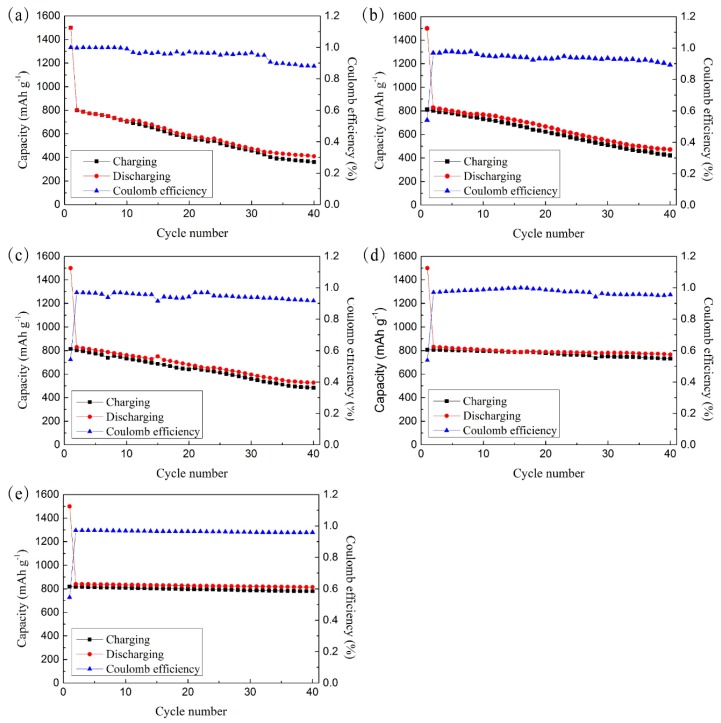
Cyclic performance and coulomb efficiency of a Li-ion battery assembled by (**a**) flat copper and CFSCC with different sizes of micro copper powders: (**b**) 0–25 μm, (**c**) 25–50 μm, (**d**) 50–75 μm, and (**e**) 75–100 μm.

Figure 3 shows the cyclic performance and coulomb efficiency of the cells assembled with either flat copper foil or CFSCC. The cells were cycled at 0.2 mA/cm^2^ between 0.02 and 1.5 V. For all samples, the discharge capacity was about 1500 mA·h/g in the first cycle, and then dropped to about 800 mA·h/g in the following cycles. In Figure 3a, the electrode with a flat copper foil current collector had a coulomb efficiency of 99.7% in the initial cycle and dropped to 88.1% after 40 cycles. Its reversible capacity sharply decreased 48.9%, from 802.5 to 409.7 mA·h/g. This degradation is related to the lithiation and delithiation of the silicon electrode. After repeating lithiation and delithiation, the volume of the silicon electrode considerably changed, which led to a poor interface contact with the current collector and larger inner resistance. Lee et al. [11]. proved that the compressive stresses of the silicon make preferential lithiation occur at free surfaces, and these mechanical interactions enhance the fracture resistance of lithiated silicon by decreasing the tensile stress concentrations in silicon structures Figure 3b–e show the performances of different-sized powder CFSCCs. The trend demonstrates that as the copper powders increase in size, the performance improves. In Figure 3e, CFSCC with a powder size of 75–100 μm in the second cycle had a capacitance of 830.54 mA·h/g and 97% coulomb efficiency. After 40 cycles, its capacitance remained at 92.2% (765.9 mA·h/g) and 95.5% of the coulomb efficiency compared with the second cycle. This phenomenon can be explained by the CFSCC constraint effect. When the silicon electrode was pasted onto the CFSCC, part of the electrode filled in the gaps among the micro copper powders, which was constrained by the powders during lithiation and delithiation. As the size of the copper powders increased, a larger portion of the electrode fills in the gap. In Figure 3e, nearly all the gaps of the electrode are filled among the micro copper powders. This phenomenon suggests that the constraint of micro copper powders on the electrode improves the cyclic performance of the silicon electrode.

**Figure 4:**
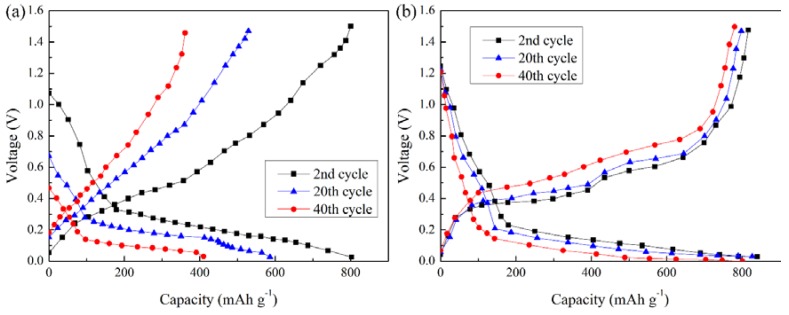
The voltage-capacity profile of a Li-ion battery assembled by a copper current collector with (**a**) flat copper and (**b**) CFSCC.

Figure 4 shows the voltage-capacity curves of the cells assembled with either a flat copper foil or CFSCC during lithiation and delithiation processes for the second, 20th, and 40th cycle. As shown in Figure 4a, as the cycle times increase, the de-lithium voltage of the electrode with a flat copper foil current collector changes greatly, suggesting that this electrode has a serious polarization. In addition, its capacity attenuates rapidly with the increased cycle times. This is due to the gradual disintegration of the electrode structure, resulting in severe electrode polarization and large electrode impedance. Compared with the electrode with a flat copper foil current collector, the electrode with CFSCC has a large Li-ion capacity, small de-intercalation voltage variation, and low electrode polarization, as shown in Figure 4b. Furthermore, the electrode with a flat copper foil had a significantly weaker voltage plateau than that with CFSCC. This difference in performance also suggests that the silicon-based anode materials have poor interface contact with the flat copper foil, whereas the electrode remains in good interface contact with CFSCC. This phenomenon is because the microspheres embedded in the electrode materials enhance the conductivity of the electrode. Even when the electrode material produces cracks owing to the desorption of Li-ion, the copper collection can still gather more electrons and avoids the polarization caused by the delay of ion diffusion to a certain extent.

### 3.3. Surface Morphology Analysis

**Figure 5:**
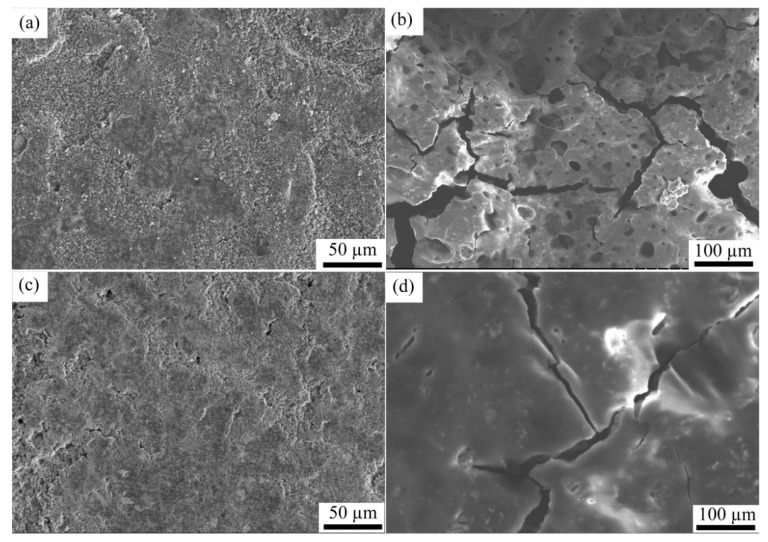
SEM micrographs of the surface morphology of the paste-like coated electrodes. Flat copper current collector (**a**) before cycling, (**b**) after 40 C-D cycles; and CFSCC (**c**) before cycling, and (**d**) after 40 C-D cycles.

Figure 5 shows the scanning electron microscopy (SEM) pictures of the electrode paste on the flat copper foil and CFSCC, before and after 40 charging-discharging (C-D) cycles. As shown in Figure 5a,c, no distinct surface morphology difference was observed between the electrode with the two different current collectors before the C-D cycles. Because the binder occupied only 10% of the electrode weight, the electrode surface was not completely smooth, but a little grainy. No fractures or voids were observed before the C-D cycles. Figure 5b,d show the surface morphology after 40 C-D cycles. After 40 C-D cycles, we observed distinct differences in the surface morphologies of the two electrodes. From Figure 5b, the electrode with the flat current collector was pulverized, and many voids appeared on the surface. This pulverization is mainly caused by the Si volume change during the lithiation and delithiation cycles; it leads to electrode detachment with the current collector, higher inner resistance, higher interfacial resistance, and decreased cyclic performance. As shown in Figure 5d, no obvious void was observed, and the electrode surface became even smoother than before. This difference in morphology shows CFSCC does have a constraining effect on the electrode volume expansion, and the electrode demonstrated little pulverization with CFSCC. Both Figure 5b,d show some cracks in the electrode, which means CFSCC cannot fully constrain the volume change of the electrode.

## 4. Conclusions

In this work, we proposed a facile sintering method to manufacture 3D CFSCC. Our concluding remarks are summarized as follows:

(1) This sintering method can be easily applied to large-scale production.
(2) The CFSCC is technically suitable for Si-based paste electrodes, which are inexpensive, and the paste-like electrode is commonly used in industrial production.
(3) Through the electrochemical test and SEM images, the CFSCC shows the ability to constrain the Si volume change and had better electric performance. The CFSCC with a powder size of 75–100 μm performed best in this study. Compared with the flat current collector, the capacitance of the electrodes with CFSCC remained as high as 92.2% of its second cycle after 40 cycles, whereas that of electrodes with the flat current collector only remained at 51.1%.
